# The Herpesvirus Nuclear Egress Complex Component, UL31, Can Be Recruited to Sites of DNA Damage Through Poly-ADP Ribose Binding

**DOI:** 10.1038/s41598-017-02109-0

**Published:** 2017-05-15

**Authors:** Maxwell R. Sherry, Thomas J. M. Hay, Michael A. Gulak, Arash Nassiri, Renée L. Finnen, Bruce W. Banfield

**Affiliations:** Department of Biomedical and Molecular Sciences, Queen’s University, Kingston, Canada

## Abstract

The herpes simplex virus (HSV) *UL31* gene encodes a conserved member of the herpesvirus nuclear egress complex that not only functions in the egress of DNA containing capsids from the nucleus, but is also required for optimal replication of viral DNA and its packaging into capsids. Here we report that the UL31 protein from HSV-2 can be recruited to sites of DNA damage by sequences found in its N-terminus. The N-terminus of UL31 contains sequences resembling a poly (ADP-ribose) (PAR) binding motif suggesting that PAR interactions might mediate UL31 recruitment to damaged DNA. Whereas PAR polymerase inhibition prevented UL31 recruitment to damaged DNA, inhibition of signaling through the ataxia telangiectasia mutated DNA damage response pathway had no effect. These findings were further supported by experiments demonstrating direct and specific interaction between HSV-2 UL31 and PAR using purified components. This study reveals a previously unrecognized function for UL31 and may suggest that the recognition of PAR by UL31 is coupled to the nuclear egress of herpesvirus capsids, influences viral DNA replication and packaging, or possibly modulates the DNA damage response mounted by virally infected cells.

## Introduction

Herpesviruses have large double stranded DNA genomes that are replicated in the nuclei of infected cells. The early stages of herpesvirus virion assembly also take place in the infected cell nucleus where newly synthesized viral genomes are packaged into preformed capsids, however, the final stages of virion maturation take place in the cytoplasm. DNA-containing capsids, also called C-capsids, have a diameter of approximately 125 nm and are too large to pass through nuclear pore complexes. The translocation of C-capsids from the nucleoplasm to the cytoplasm begins with the recruitment of C-capsids to the inner nuclear membrane (INM) followed by their budding at the INM into the perinuclear space to form transient perinuclear virions. Finally, the fusion of the perinuclear virion envelope with the outer nuclear membrane releases the C-capsid into the cytoplasm. This process is collectively referred to as nuclear egress and has been the subject of intense investigation and several recent comprehensive reviews^1–3^. Subsequent to nuclear egress, cytoplasmic C-capsids acquire a final envelope by budding into membranes of the trans Golgi network, or possibly at a late, or recycling, endosomal compartment^4, 5^. Vesicles containing enveloped virions then traffic to the cell surface where they fuse with the plasma membrane to release virions into the extracellular environment.

The herpesvirus nuclear egress complex is comprised of two conserved viral proteins encoded by the orthologs of the herpes simplex virus (HSV) genes *UL31* and *UL34*. UL34 is a type II membrane protein that localizes to the endoplasmic reticulum and nuclear membranes whereas UL31 is a soluble nucleoplasmic phosphoprotein. UL31 and UL34 interact directly and, when these proteins are co-expressed, UL31 can also localize to the nucleoplasmic face of the INM via its interaction with UL34^6, 7^. In the absence of other viral proteins, UL31 and UL34 have the remarkable capacity to direct the formation of vesicles from the INM in a process that has been elegantly recapitulated *in vitro* using purified proteins and synthetic membranes^8, 9^. Recently, Lorenz and colleagues demonstrated that pseudorabies virus (PRV) UL31 was sufficient to direct vesicle formation and scission *in vitro* if it was artificially tethered to membranes suggesting that a key function of UL34 in nuclear membrane remodeling is recruitment of UL31 to the membrane^10^. As UL31 can also bind to nuclear capsids, one model for capsid recruitment to the INM posits that UL31 bound to C-capsids facilitates the recruitment of the C-capsid to the INM that contains UL34 where the interaction between UL31 and UL34 facilitates primary envelopment^11–13^. Whereas the C-terminus of UL31 orthologs contains four conserved regions, the N-terminus of UL31 is much more variable in sequence between viruses^14^. Despite this variability the N-terminus of the HSV-1 UL31 ortholog plays a critical role in virus propagation^15, 16^. A bipartite nuclear localization signal as well as several serine residues that can be phosphorylated by the viral serine/threonine kinase Us3 are located within the N-terminus of HSV-1 UL31 and are thought to regulate UL31 activity^15, 16^. The authors of a recent study have suggested that the N-terminus of HSV-1 UL31 may function as a switch that regulates UL31/UL34 interactions necessary for the primary envelopment of capsids^15^.

The UL31/UL34 complex plays a critical, although not strictly essential, role in the egress of C-capsids from the nucleus of infected cells insofar as deletion of HSV-1 UL31, for example, results in a 3–4 log reduction in the production of infectious virus in many, but not all, cell types^17, 18^. Early studies demonstrated that this replication deficiency was not only accompanied by the accumulation of C-capsids in the nucleus of infected cells but also with reductions in virus DNA replication and decreased packaging of viral genomes into capsids^17^. These findings suggested additional functions for UL31 beyond its role nuclear egress. In support of these initial studies, Dembowski and DeLuca found HSV-1 UL31 in association with newly replicated HSV-1 DNA^19^. Analysis of UL31 orthologs from the betaherpesvirus MCMV^20^ and the gammaherpesvirus EBV^21^ have also revealed distinct roles for these proteins in both packaging of viral DNA into capsids and nuclear egress of capsids. A role for UL31 in promoting HSV-1 gene expression at early times after infection has been described and correlates with the failure of cells infected with UL31 null viruses to activate NF-kB and the MAP kinase, JNK, both of which have been implicated in promoting virus infection^22, 23^.

In this study we demonstrate that the N-terminus of HSV-2 UL31 contains sequences that mediate the localization of UL31 to the nucleus as well as poly(ADP-ribose) (PAR) binding activity. PAR binding proteins bind non-covalently to PAR, a polymer of ADP ribose subunits synthesized by PAR polymerases (PARPs) using NAD+ as a substrate (reviewed in ref. 24). Poly(ADP-ribosylation) (PARylation), the covalent attachment of PAR to proteins via Glu, Asp and Lys residues, can range from two to approximately 200 ADP-ribose units in length. These polymers may be linear or branched depending on the PARP catalyzing the polymerization reaction. Seventeen PARPs have been identified in the human genome, however, the precise functions of many of these enzymes remains obscure. Intriguingly, five of these PARPs are undergoing rapid evolution suggesting a role for PARylation and PAR recognition in virus-host interactions^25^. PARylation of proteins occurs rapidly, yet transiently, in response to a variety of cellular stresses and can be reversed through the action of enzymes such as PAR glycohydrolase (PARG). The most well studied PARPs, PARP1 and PARP2 (PARP1/2), function as sensors of DNA damage (reviewed in ref. 26). Upon direct binding to damaged DNA PARP1/2 undergo auto-PARylation, which coincides with PARylation of other proximal proteins. The resulting foci of PARylated proteins serve as a platform for the recruitment of additional PAR binding proteins involved in the coordination and execution of DNA damage repair. These findings may suggest that the recognition of PAR by UL31 is coupled to the nuclear egress of herpesvirus capsids, influences viral DNA replication and packaging, or possibly modulates the DNA damage response mounted by virally infected cells.

## Results

### HSV-2 UL31 is recruited to sites of DNA damage

**Figure 1:**
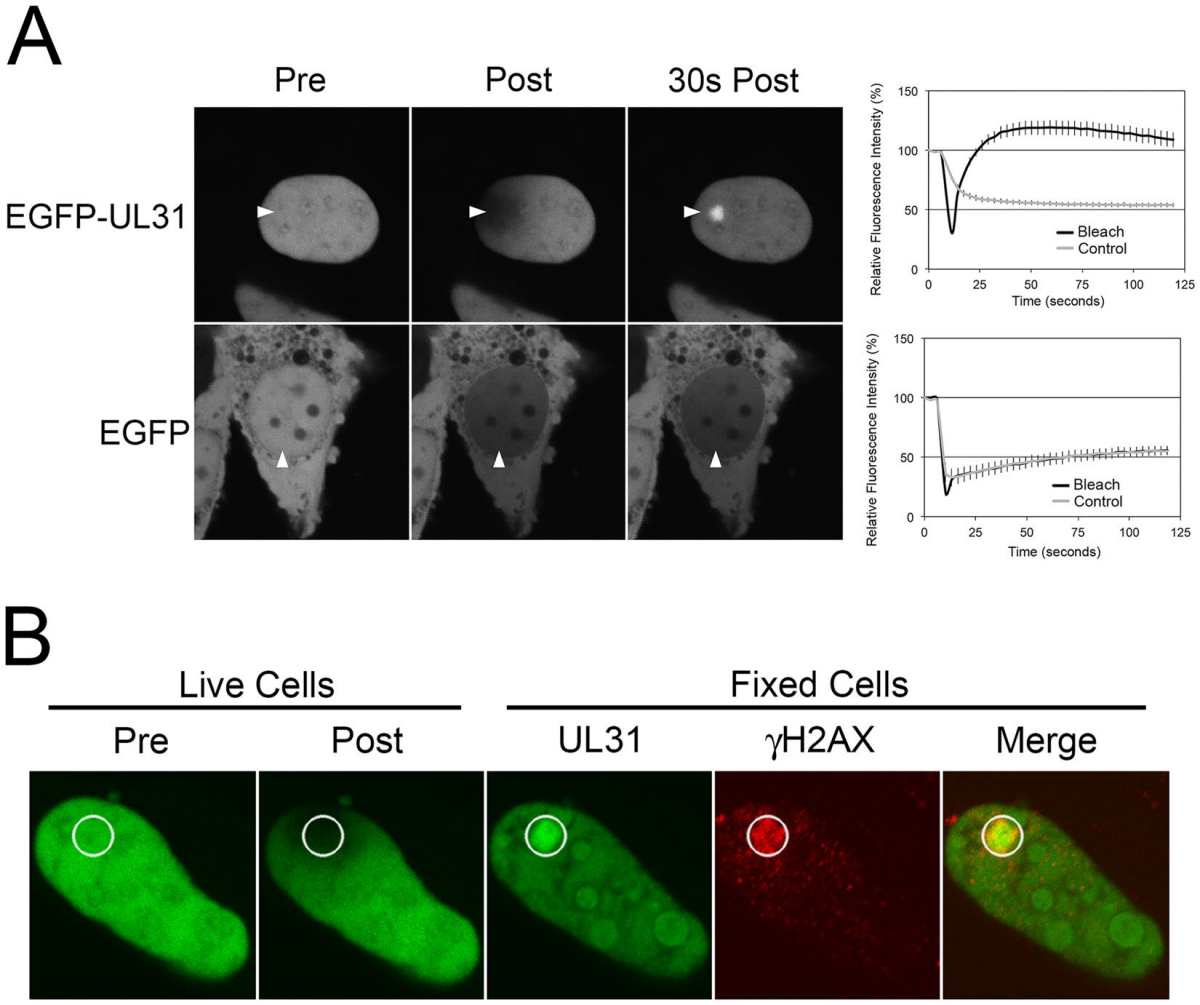
UL31 accumulates at sites of laser microirradiation-induced DNA-damage. (**A**) Representative images of live HeLa cells transfected with EGFP or EGFP-UL31 expression plasmids immediately before (Pre), immediately after (Post), and 30 seconds after the areas indicated by the arrowheads were microirradiated with a 405 nm laser using an Olympus FV1000 laser-scanning confocal microscope. The graphs display quantitation of the average fluorescence intensity of the microirradiated area (Bleach), as well as control distal areas (Control), over time for n = 20 cells in four independent experiments for EGFP-UL31 and for n = 22 cells in three independent experiments for EGFP. Error bars are standard error of the mean. (**B**) Images of a live HeLa cell transfected with EGFP-UL31 on grid-bottom dishes immediately before, and immediately after the indicated area (white circle) was microirradiated with a 405 nm laser using an Olympus FV1000 laser-scanning confocal microscope (Live). Cells were immediately fixed and stained with anti-γH2AX antibody followed by staining with Alexa 568 conjugated donkey anti-mouse secondary antibody. Microirradiated cells were relocated and images captured by confocal microscopy (Fixed). Image is representative of n = 8 cells analyzed in two independent experiments.

We noted that exposure of cells expressing EGFP-UL31 to localized intense 405 nm laser microirradiation within the nucleus resulted in the rapid and preferential recruitment of EGFP-UL31 to these sites during fluorescence recovery after photobleaching assays (Fig. 1A). By contrast, localized irradiation within the nuclei of cells expressing non-fused EGFP did not result in preferential recovery of fluorescence to the site of irradiation. As intense 405 nm laser exposure results in DNA damage, including double-stranded DNA breaks^27^, we hypothesized that UL31 was being recruited to sites of DNA damage. To test this, cells expressing EGFP-UL31 growing on glass bottom dishes etched with an alphanumeric grid were exposed to localized 405 nm irradiation then immediately fixed and stained for γH2AX, a marker for sites of double-stranded DNA breaks^28^. The alphanumeric grid enabled re-identification of microirradiated cells after immunohistochemisty. These experiments revealed that γH2AX reactivity co-localized with EGFP-UL31 at the irradiation site (Fig. 1B). These findings support the notion that UL31 can be specifically recruited to sites of DNA damage.

### HSV-2 UL31 recruitment to sites of DNA damage requires N-terminal sequences but not the ability to interact with UL34

**Figure 2:**
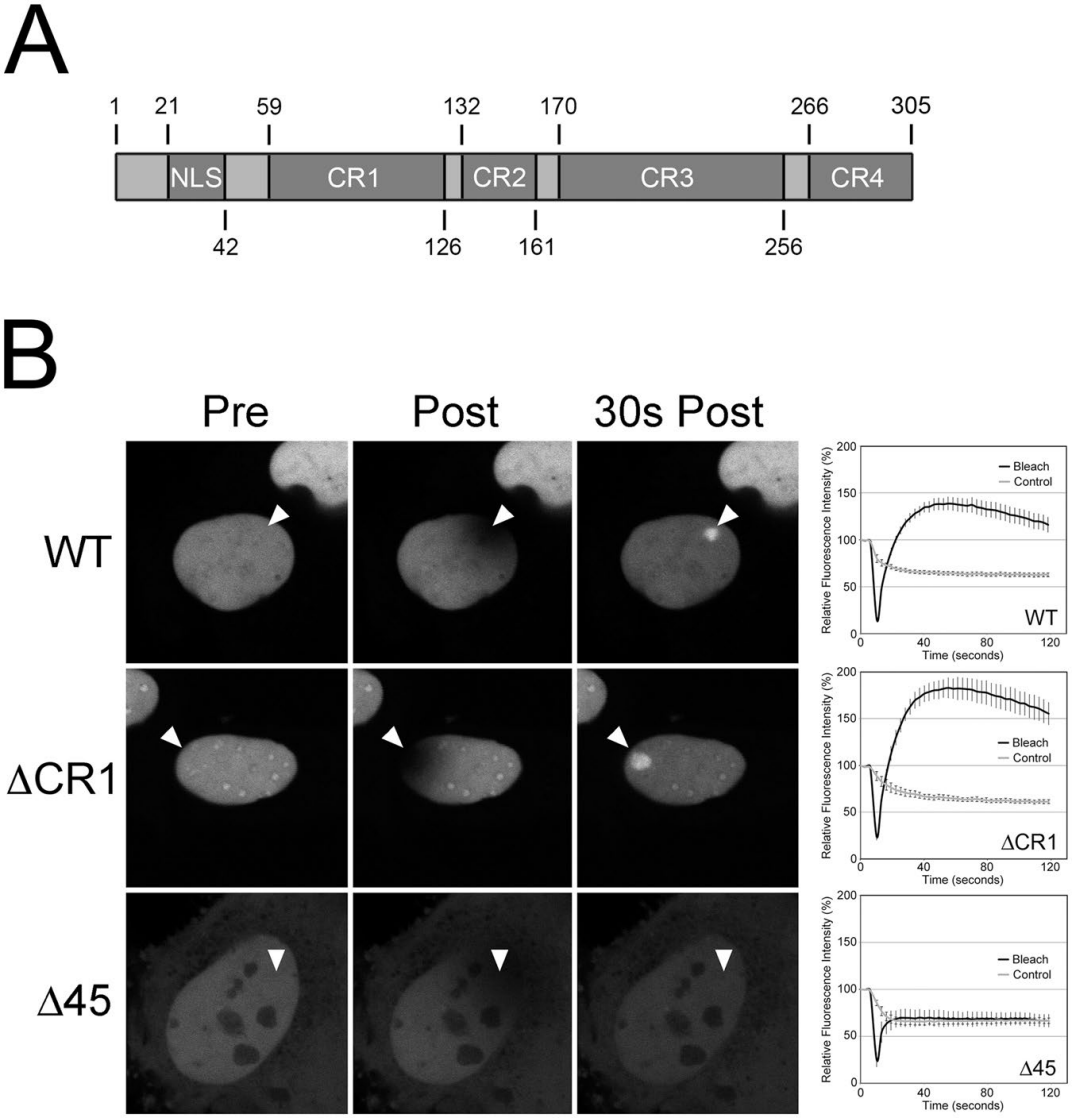
Recruitment of UL31 to sites of laser microirradiation requires its N-terminal 45 amino acids but not CR1. (**A**) Cartoon of HSV-2 UL31 identifying key features of the molecule including the position of the bipartite nuclear localization signal (NLS) as well as the positions of conserved regions (CR) 1 through 4. (**B**) Images of live HeLa cells expressing wild type EGFP-UL31 (WT), EGFP-UL31 lacking the CR1 region required for UL34 binding (ΔCR1), or EGFP-UL31 lacking its N-terminal 45 amino acids (Δ45) acquired immediately before (Pre), immediately after (Post), and 30 seconds after the areas indicated by arrowheads were microirradiated using an Olympus FV1000 laser-scanning confocal microscope. The graphs display quantitation of the average fluorescence intensity of the irradiated area (Bleach), as well as distal control areas (Control), over time. Images shown are representative of n = 12 cells analyzed in two independent experiments. Error bars are standard error of the mean (n = 12 per condition).

**Figure 3:**
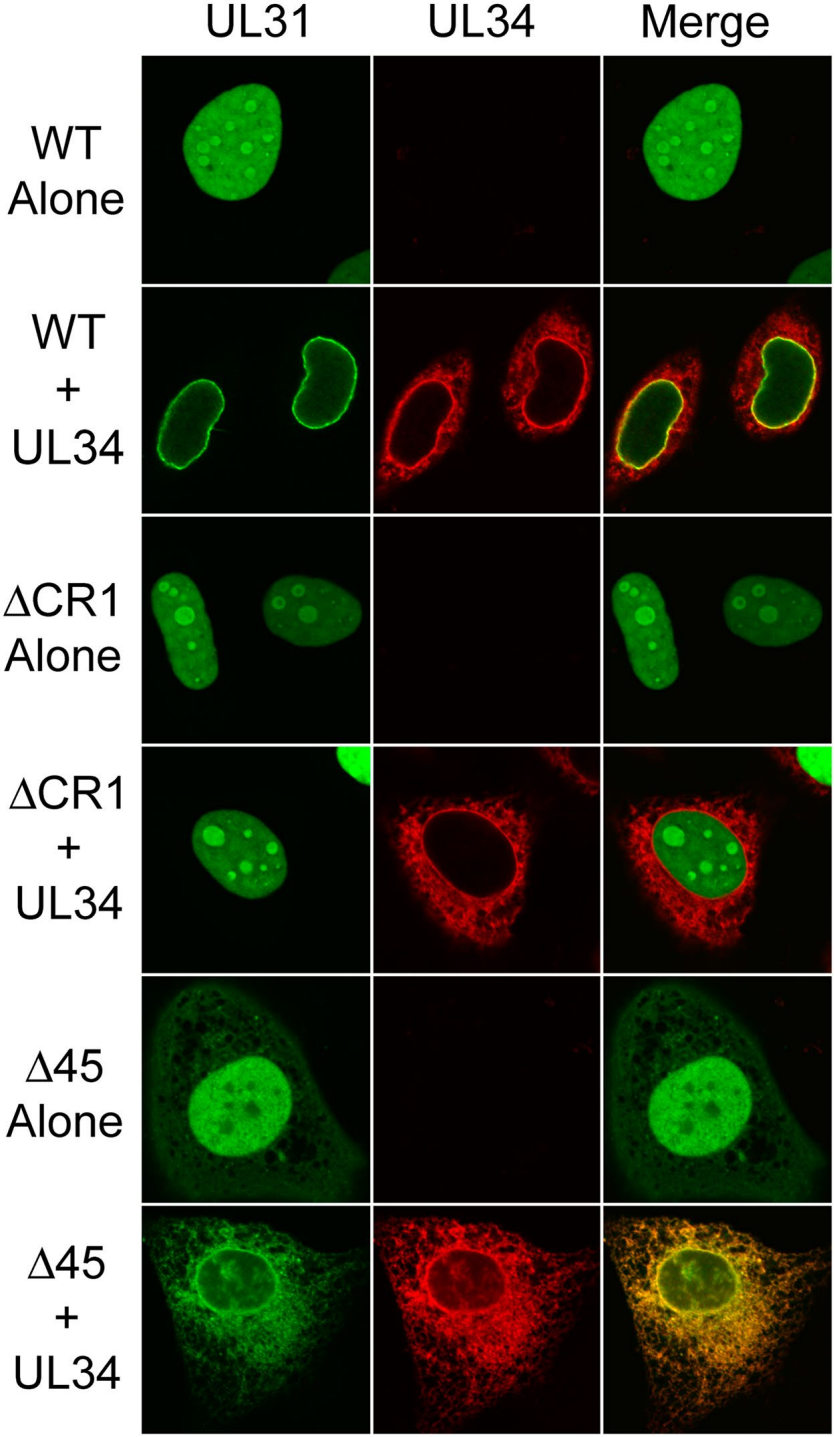
Recruitment of UL31 to sites of laser microirradiation is independent of its ability to interact with UL34. HeLa cells were transfected or co-transfected with plasmids encoding the EGFP-tagged WT, ΔCR1 or Δ45 UL31 and, where indicated, FLAG-tagged UL34. At 16 h post transfection, cells were fixed and stained with anti-FLAG antibody and Alexa 568 conjugated secondary antibody (red signal). Representative images acquired by confocal microscopy are shown.

To begin to define the requirements for UL31 recruitment to DNA damage, two EGFP-UL31 mutants were constructed. ΔCR1 has a deletion of the UL34 binding domain (residues 59–126)^14^ and Δ45 contains a deletion of the N-terminal 45 amino acids. The ΔCR1 mutant maintained the ability to be recruited to sites of DNA damage (Fig. 2) however, as expected, it failed to co-localize with UL34 at the INM (Fig. 3). By contrast, Δ45 was not recruited to sites of DNA damage (Fig. 2) and retained the ability to co-localize with UL34 (Fig. 3). These data indicate that UL31 recruitment to sites of DNA damage can be separated from its ability to interact with UL34 and that the N-terminus of UL31 is required for its recruitment to DNA damage. We also noted that the Δ45 mutant displayed increased cytoplasmic localization compared to the almost exclusively nuclear WT and ΔCR1 mutant localization patterns. This was particularly striking when Δ45 was co-expressed with UL34 where these molecules co-localized at both the nuclear rim and at cytoplasmic membranes. Notably, unlike the WT and the ΔCR1 mutant the Δ45 mutant failed to localize to nucleoli. These findings imply that sequences contained within the N-terminal 45 residues of HSV-2 UL31 influence both its nuclear and sub-nuclear localization.

### The N-terminus of UL31 is critical for UL31 nuclear localization and is sufficient for recruitment to sites of DNA damage

**Figure 4:**
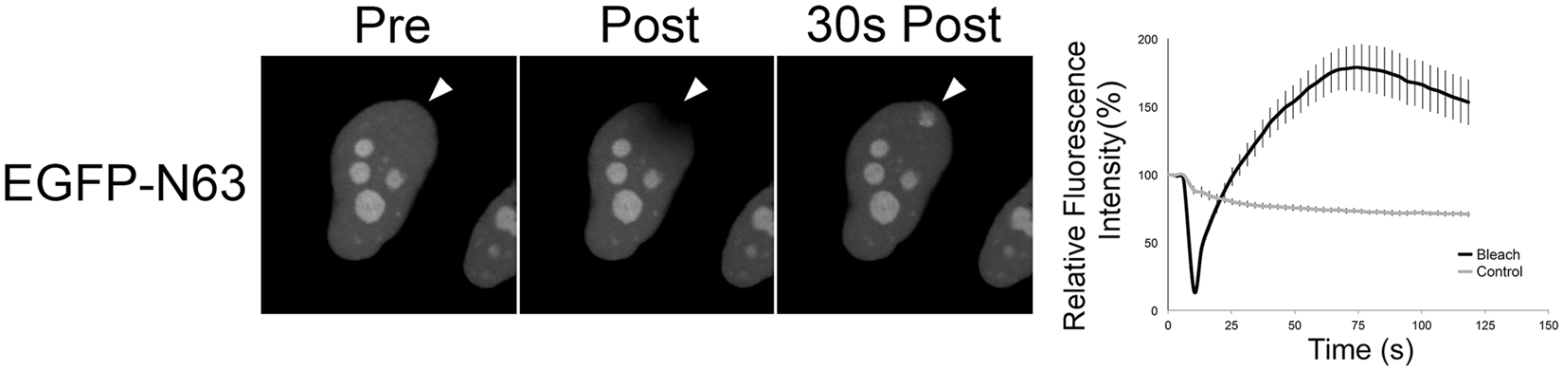
The N-terminal 63 amino acids of UL31 are sufficient to recruit EGFP to sites of laser microirradiation. Images of a live HeLa cell transfected with EGFP-N63 immediately before (Pre), immediately after (Post), and 30 seconds were acquired after the area indicated by the arrowhead was irradiated using an Olympus FV1000 laser-scanning confocal microscope. Images shown are representative of n = 12 cells analyzed in two independent experiments. Error bars are standard error of the mean (n = 12).

**Figure 5:**
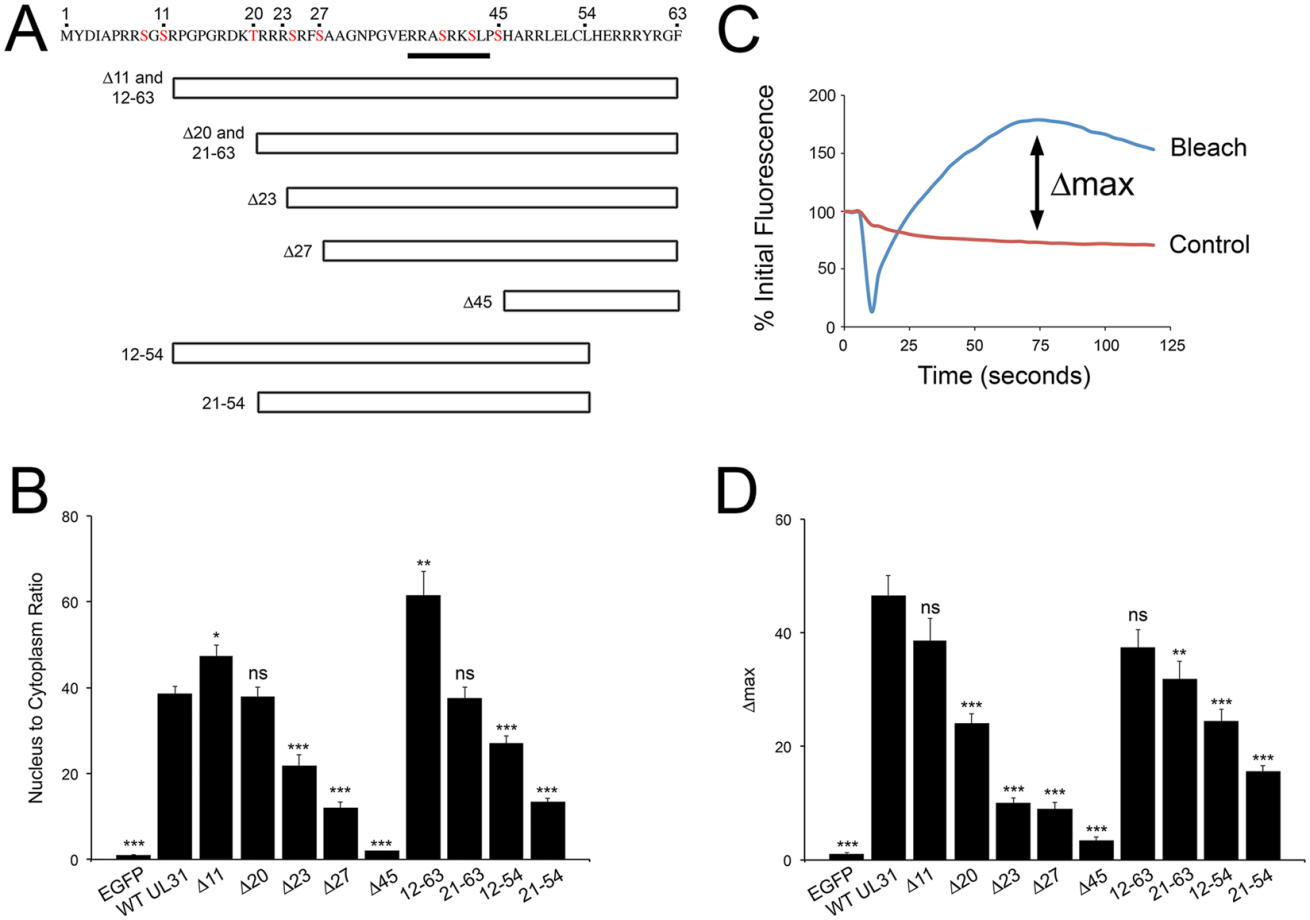
Identification of regions of UL31 required for nuclear localization and recruitment to sites of laser microirradiation. (**A**) Sequence of the N-terminal 63 amino acids of HSV-2 UL31. Serine (S) and threonine (T) residues highlighted in red are predicted to be modified by Us3^16^. Underlined residues are 75% similar to a canonical poly-ADP-ribose binding motif. Various C-terminal and N-terminal truncations of UL31 analyzed in these experiments are shown. (**B**) Images of live HeLa cells transfected with plasmids expressing the indicated EGFP tagged protein were captured using an Olympus FV1000 laser-scanning confocal microscope over the course of two independent experiments with n ≥ 7 cells/experiment. The relative fluorescence intensity in the nucleus and the cytoplasm was determined using Fluoview software version 1.7.3.0 and the nucleus:cytoplasm ratio calculated. Error bars represent standard error of the mean. Using an unpaired *t* test the nucleus to cytoplasm ratio of WT UL31 was compared to the other proteins (ns = not significant, *p < 0.05, **p < 0.01, ***p < 0.001). (**C**) We define Δmax as the largest positive difference between the fluorescence intensity of the laser microirradiated region (Bleach) and a control region (Control) over the course of the experiment. (**D**) The Δmax of the various UL31 constructs. HeLa cells transfected with plasmids expressing the indicated protein were microirradiated as described above and Δmax was calculated for each construct. n ≥ 7 cells/experiment in two independent experiments for each construct analyzed. Error bars represent standard error of the mean. Using an unpaired *t* test the Δmax of WT UL31 was compared to the other proteins (ns = not significant, *p < 0.05, **p < 0.01, ***p < 0.001).

The data so far suggested that the N-terminus of UL31 was required for its recruitment to damaged DNA. We next tested if the N-terminal variable domain of UL31^29^ was sufficient for this activity by fusing the N-terminal 63 amino acids to EGFP (Fig. 4). EGFP-N63 was preferentially and rapidly recruited to sites of microirradiation. Moreover, the N-terminal 63 residues was sufficient to restrict EGFP localization to the nucleus further suggesting that this region contains a functional nuclear localization signal (NLS). Additionally, EGFP-N63 localized to nucleoli. The N-terminal 63 amino acids of HSV-2 UL31 contains several notable features including serine and threonine residues that are known to be phosphorylated by the virus serine/threonine kinase Us3 in HSV-1 UL31 ΔCR1^16^ as well as numerous clustered basic residues that could potentially function as either a monopartite, or bipartite, NLS^29^ (Fig. 5A). A series of truncated EGFP-UL31 fusions were designed based on the location of serine, threonine and basic residues, and analyzed for their ability to localize to the nucleus and to be recruited to sites of microirradiation. Importantly, the expression of each of these proteins was also verified by Western blotting of transfected HeLa cell lysates where the expression of proteins of predicted mobility and negligible degradation was confirmed (data not shown).

HeLa cells were transfected with the indicated EGFP-UL31 expression plasmids and the fluorescence intensity of EGFP measured in the nucleoplasm and cytoplasm of individual living cells. The data are displayed as the nucleus:cytoplasm ratio (N/C) (Fig. 5B). The N/C of unfused EGFP was 1.04 (+/−0.01), consistent with the ability of EGFP to diffuse freely between the nucleus and cytoplasm. By contrast, the N/C of EGFP fused to WT UL31 was 38.5 (+/−1.9) indicating the presence of UL31 sequences that promote nuclear localization. Whereas deletion of 11 N-terminal residues of UL31 significantly promoted nuclear localization, elimination of 45 N-terminal residues almost abrogated nuclear localization (N/C = 2.0 +/− 0.1). The data also indicate that the three arginine residues located at positions 21–23, residues 24–27 as well as the basic stretch of amino acids between 54 and 63 also contribute to UL31 nuclear localization. However, because the Δ45 mutant lost preferential nuclear localization these findings suggest that the region between residues 54 and 63 is not sufficient to promote UL31 localization to the nucleus.

We next examined the ability of these mutant forms of UL31 to be recruited to sites of DNA damage. To simplify comparisons of these data, Δmax values were determined for each UL31 mutant construct. We define Δmax as the maximum positive difference observed in relative fluorescence intensity between the microirradiated region and a non-irradiated control region within the same nucleus (Fig. 5C). Unfused EGFP had a Δmax of 1.1(+/−0.3) indicating that there is no preferential recruitment of EGFP to sites of microirradiation (Fig. 5D). By contrast, EGFP fused to WT UL31 had a Δmax of 46.6 (+/−3.5). Whereas deletion of the N-terminal 11 amino acids of UL31 had little effect on Δmax compared to WT UL31, deletion of 20 amino acids significantly reduced Δmax suggesting that features between residues 12 and 20 contribute to UL31 recruitment to sites of DNA damage. Removal of three arginine residues (21–23) resulted in additional significant reduction in Δmax values. To explore the C-terminal boundary of this UL31 activity a stretch of predominantly basic residues (54–63) were removed and resulted in significant reductions in Δmax. However, despite reductions in Δmax, residues 21–54 when fused to EGFP were sufficient to yield a Δmax 14-fold higher than EGFP alone indicating that significant amount of recruitment activity remained within these sequences. These data suggest that multiple features within the N-terminus of UL31 contribute to its recruitment to sites of DNA damage and that sequences contained within residues 21–54 are sufficient to impart significant activity upon EGFP.

### UL31 localization to sites of DNA damage is negatively regulated by Us3

**Figure 6:**
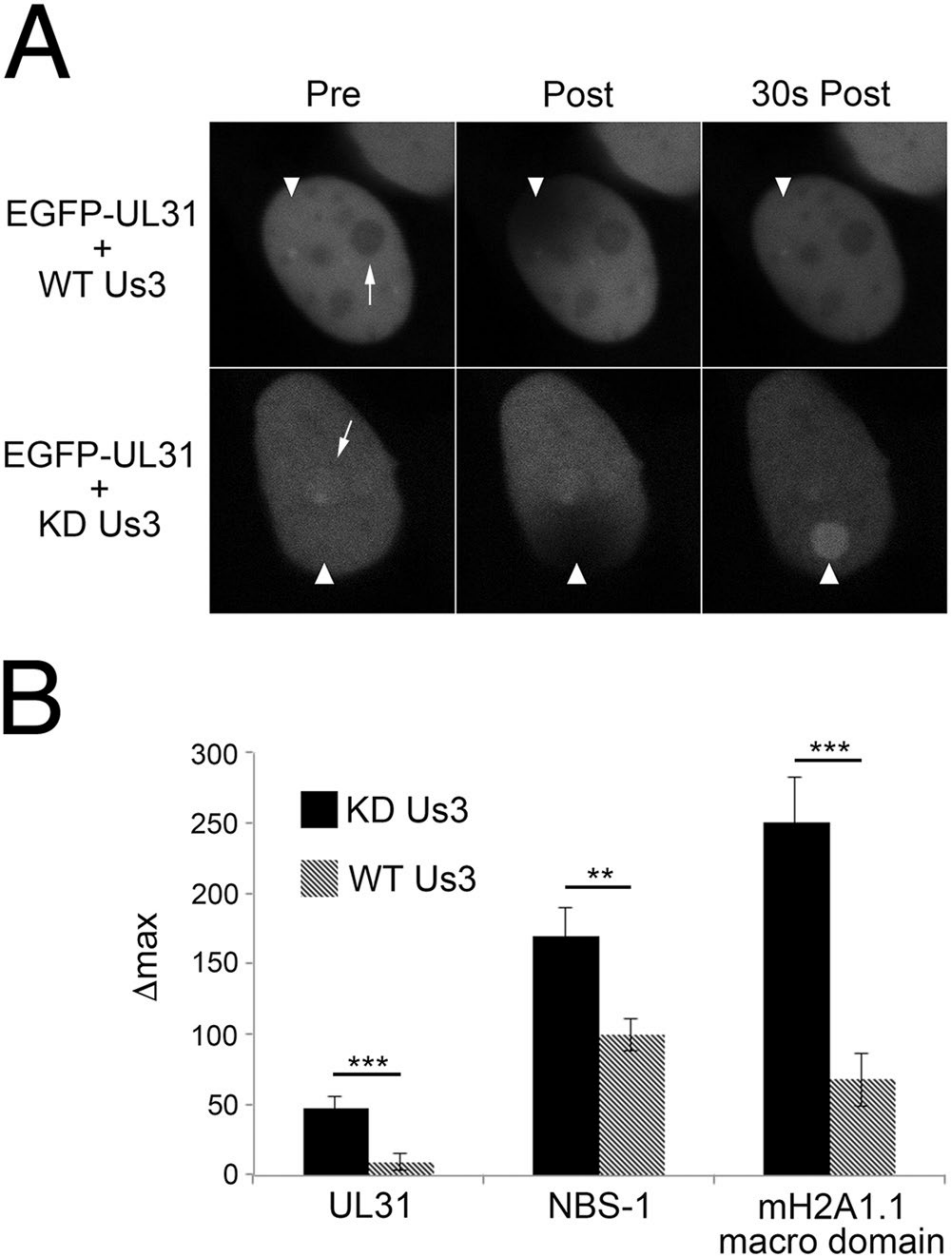
The effect of Us3 on the recruitment of UL31 to sites of DNA damage. (**A**) HeLa cells co-expressing EGFP-UL31 with either WT Us3 or kinase dead (KD) Us3 were subjected to laser microirradiation. Images are from immediately before (Pre), immediately after (Post) and 30 seconds after microirradiation. Arrows denote the location of nucleoli. (**B**) Δmax values from microirradiated cells expressing EGFP-UL31, NBS1 and H2A1.1 macro domain in the presence of WT or KD Us3. Δmax was calculated from n ≥ 14 cells for each co-transfection condition from two independent experiments. Error bars represent standard error of the mean. Using an unpaired *t* test the Δmax of KD Us3 co-expressing cells was compared to cells co-expressing WT Us3 (**p < 0.01, ***p < 0.001).

As noted above, the N-terminus of UL31 contains several serine residues that are likely phosphorylated by the viral serine/threonine kinase Us3^16^. It was therefore of significant interest to determine whether Us3 could regulate UL31 recruitment to sites of laser microirradiation. HeLa cells were co-transfected with EGFP-UL31 and either WT Us3 or a kinase dead (KD) Us3 expression plasmids (Fig. 6A). To control for non-specific effects of Us3 expression on the recruitment of proteins to sites of DNA damage two cellular DNA damage response proteins fused to EGFP, NBS-1 and the H2A1.1 macro domain, were included in this analysis. Results were quantified by measuring Δmax (Fig. 6B). Co-expression of KD Us3 with UL31, NBS-1 and the H2A1.1 macro domain resulted in robust recruitment of these proteins to sites of DNA damage, whereas co-expression of WT Us3 prevented their ability to be recruited to damaged DNA. While these data indicate that Us3 kinase activity prevents UL31 recruitment to sites of DNA damage, this effect was not specific to UL31 raising the possibility that phosphorylation of one or more cellular Us3 targets has a more general effect on protein recruitment to damaged DNA. Interestingly, co-expression of WT Us3 with UL31 prevented its localization to nucleoli similar to what was observed with the Δ45 mutant (Fig. 2) suggesting that Us3 phosphorylation of serine residues in the N-terminus of UL31 modulate UL31 sub-nuclear localization.

### Recruitment of UL31 to sites of DNA damage is insensitive to ataxia telangiectasia mutated (ATM) inhibition

**Figure 7:**
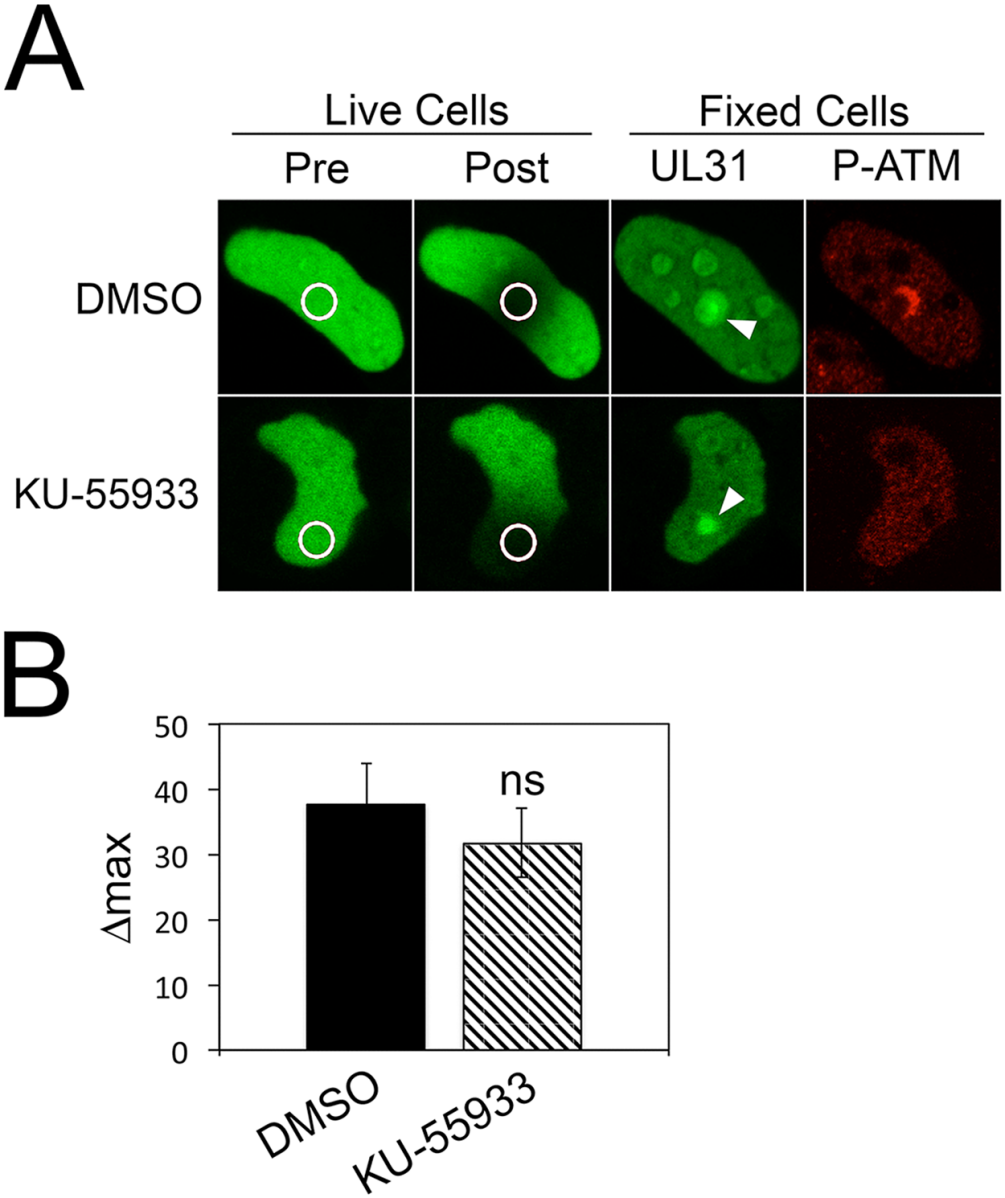
UL31 recruitment to sites of laser microirradiation is not prevented by ATM inhibition (**A**) HeLa cells on grid-bottom dishes expressing EGFP-UL31 were treated with 0.1% DMSO (vehicle) or 10 μM KU-55933, an ATM inhibitor, two hours prior to imaging. Images of cells were captured immediately before (Pre), and immediately after (Post) the areas indicated by the white circles were microirradiated using an Olympus FV1000 laser-scanning confocal microscope. Cells were immediately fixed and stained with anti-γH2AX antibody followed by an Alexa 568 conjugated secondary antibody (red signal). Microirradiated cells were relocated and images captured by confocal microscopy. Representative images of 4 cells/condition in two independent experiments are shown. (**B**) Δmax of DMSO and KU-55933 treated cells was determined as described above (n = 8 cells/condition). Error bars represent standard error of the mean. Using an unpaired *t*-test the Δmax of DMSO treated cells was compared to KU-55933 treated cells and found not to be significant (ns).

A key component of the early cellular response to DNA damage is the MRN complex composed of Mre11, Rad50 and NBS-1, which acts as a sensor of double-stranded DNA breaks (reviewed in ref. 30). This complex recruits and tethers the primary signal kinase, ATM, to the site of the DNA break. Once activated, ATM mediates the phosphorylation of proteins, including ATM itself, which act as signal amplifiers and recruit molecules to the DNA break that facilitate repair. We explored the possibility that UL31 recruitment to sites of DNA damage was dependent upon ATM activation by utilizing the ATM specific inhibitor KU-55933^31^. Whereas ATM phosphorylation was readily observed at sites of microirradiation in cells treated with vehicle alone (0.1% DMSO), treatment of cells with 10 µM KU-55933 prevented ATM phosphorylation at these sites (Fig. 7A). ATM inhibition, however, was unable to prevent UL31 recruitment to sites of DNA damage (Fig. 7A,B).

### Recruitment of UL31 to sites of DNA damage is prevented by inhibition of PARP1/2 and stimulated by inhibition of PARG

**Figure 8:**
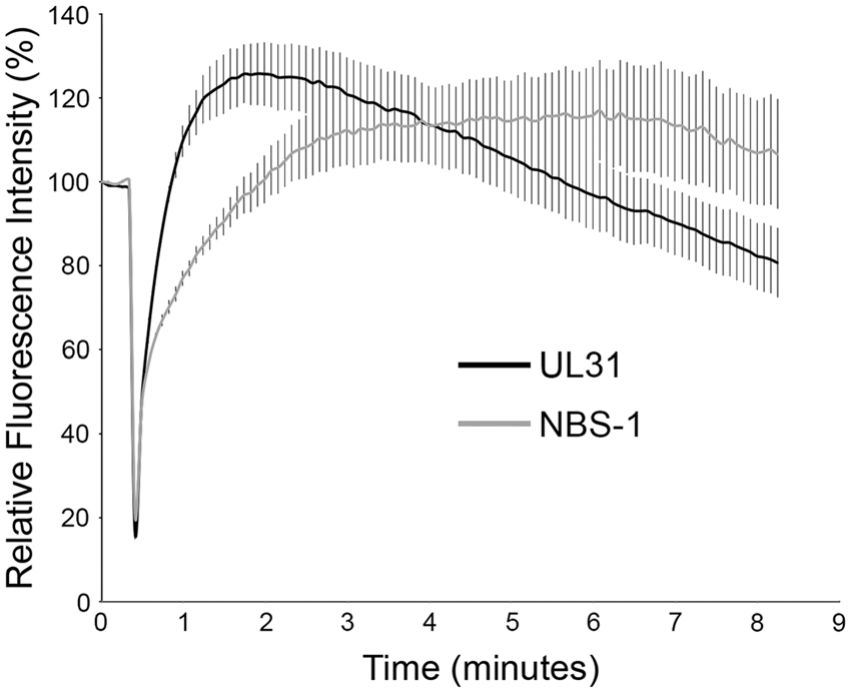
Comparative rates of recruitment of UL31 and NBS1 to sites of laser microirradiation. Nuclei of HeLa cells expressing either WT EGFP-UL31 (n = 4) or NBS-1 (n = 5) were subjected to localized microirradiation as described for Fig. 1 except that a total of 100 frames were captured at a rate of 0.2 frames per second. The graph displays the average relative fluorescence intensity of the irradiated area over time. Error bars represent standard error of the mean.

A number of cellular proteins have been identified that are recruited to damaged DNA and the kinetics of their recruitment have been examined in detail^32^. To provide mechanistic clues to how UL31 is recruited to damaged DNA we measured UL31 recruitment kinetics in comparison to those of NBS-1 (Fig. 8). Similar to what has been reported previously^33^, we found NBS-1 recruitment reached maximal levels 346 seconds after induction of DNA damage and was maintained at constant levels at the break site for the duration of the experiment. By contrast, the recruitment kinetics of UL31 was very different than that seen for NBS-1. Maximal recruitment of UL31 occurred at 95 seconds post irradiation and then began to steadily disappear from the DNA damage site over the course of the experiment. The kinetics of UL31 recruitment to damaged DNA closely resembled those reported for PARP1^34^.

**Figure 9:**
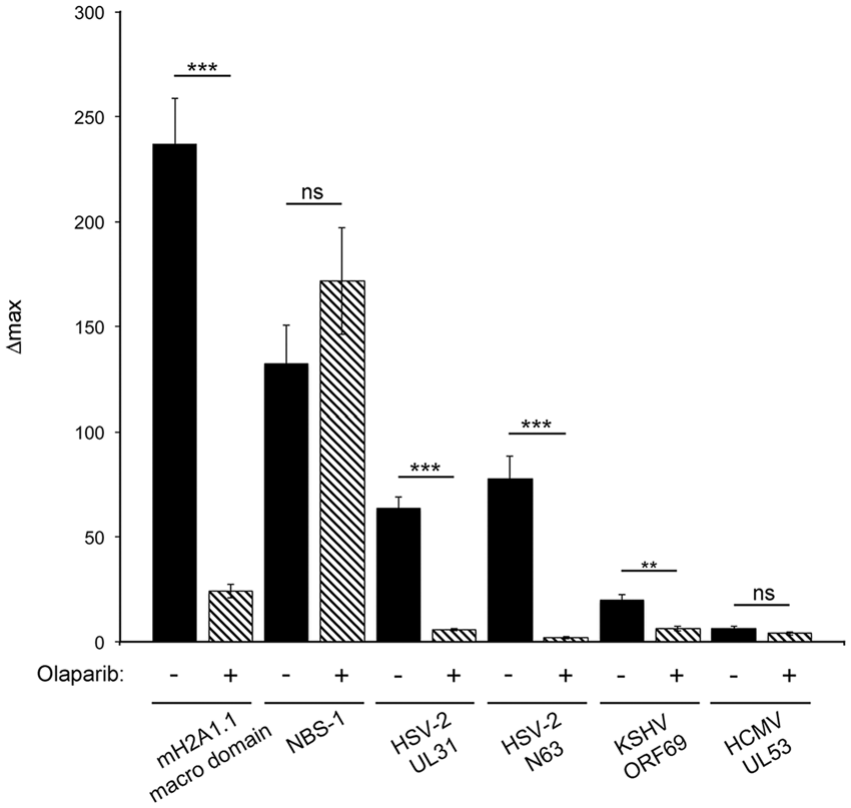
UL31 recruitment to sites of laser microirradiation requires PARP1/2 activity. HeLa 0.1% DMSO (vehicle) or 10 nM olaparib, a PARP1/2 inhibitor, one hour prior to imaging. Δmax values of 0.1% DMSO (−) and 10 nM olaparib treated (+) cells expressing the indicated proteins was determined as described above in Fig. 5. n ≥ 6 cells/experiment in two to four independent experiments for each construct analyzed. Error bars represent standard error of the mean. Using an unpaired *t* test the Δmax of DMSO treated cells was compared to olaparib treated cells (ns = not significant, **p < 0.01, ***p < 0.001).

Between UL31 residues 37 and 44 is a sequence, RASRKSLP, that has 75% similarity to the PAR binding motif (PBM) (Fig. 5A)^35^. The presence of a putative PBM within UL31 is significant as PARylation is a posttranslational modification performed by PARP1/2 that is stimulated by, and at, sites of DNA damage where it recruits PAR binding proteins that coordinate chromatin modification and repair of damaged DNA^26^. To test if UL31 recruitment to sites of DNA damage was dependent upon PARP1/2 activity we utilized the potent inhibitor of PARP1/2, olaparib (Fig. 9)^36^. As a positive control, we examined the ability of olaparib to prevent the recruitment of the mH2A1.1 macrodomain, a known PAR binding molecule^37^, to sites of DNA damage and found that it was potently inhibited. As a negative control, we examined the effects of olaparib on recruitment of the MRN complex component NBS-1 to sites of DNA damage and found that Δmax was unaffected by olaparib. Similar to the mH2A1.1 macrodomain, UL31 recruitment to sites of DNA damage was eliminated by exposure of cells to olaparib indicating that PARP1/2 activity is required for UL31 recruitment.

**Figure 10:**
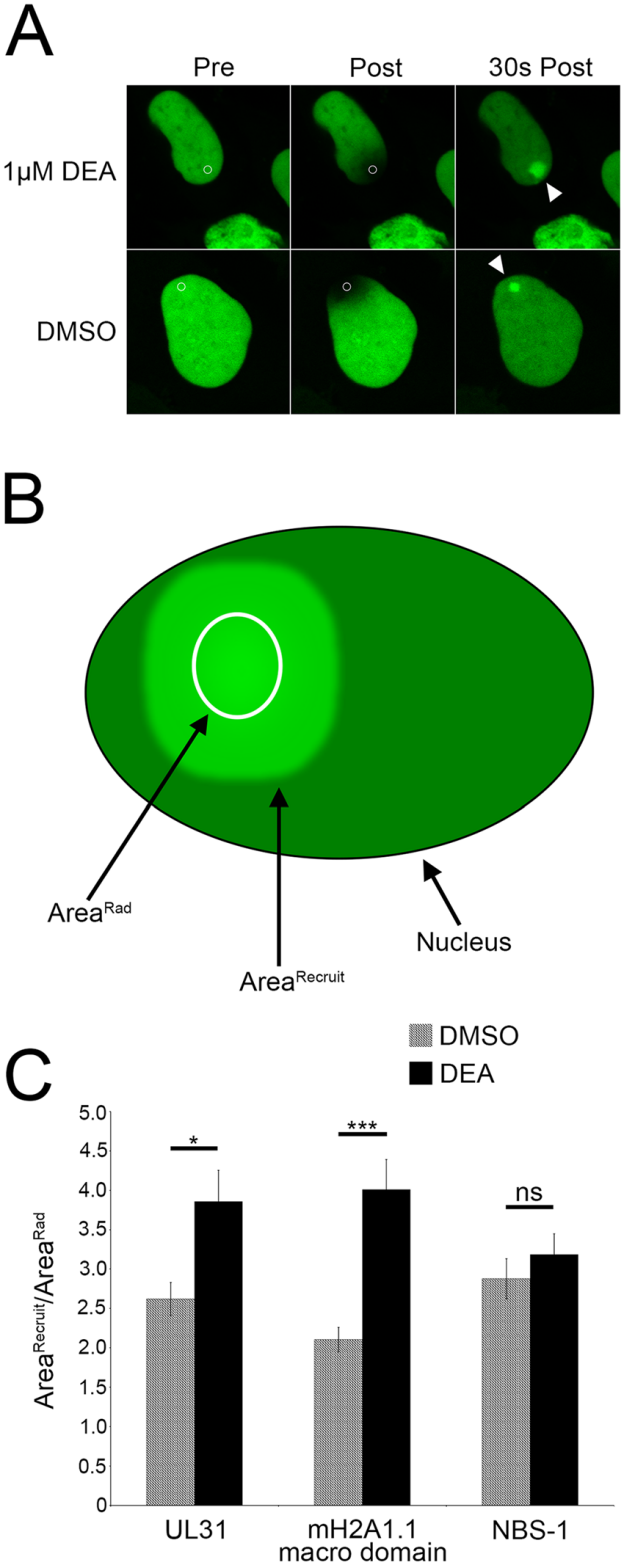
Inhibition of PARG results in enhanced recruitment EGFP-UL31 to sites of laser microirradiation. (**A**) Cells expressing EGFP-UL31 were treated with 0.1% DMSO (vehicle) or 1 μM DEA, a PARG inhibitor, two hours prior to imaging. Images of cells were captured immediately before (Pre), immediately after (Post) and 30 seconds after the region within the white circle was microirradiated using a 405 nm laser line. The intensity of radiation and size of the area of irradiation were maintained between all cells irradiated. (**B**) We define the ratio of areas, Area^Recruit^/Area^Rad^, as the area of protein recruitment at Δmax (Area^Recruit^) divided by the initial area that was microirradiated (Area^Rad^). (**C**) Graph of the ratio of areas of UL31, NBS1 and H2A1.1 macro domain in DMSO (vehicle) and 1 µM DEA treated cells. Area^Recruit^/Area^Rad^ was calculated for each construct (n ≥ 11) from two independent experiments. Error bars represent standard error of the mean. (ns = not significant, *p < 0.05, ***p < 0.001).

Taking a reciprocal approach to the experiments described above, we postulated that inhibition of PARG, a key enzyme in dismantling PAR chains, might enhance UL31 recruitment to sites of DNA damage. Representative images from microirradiation experiments performed on cells transfected with EGFP-UL31 and incubated with the PARG inhibitor DEA or DMSO (vehicle control) are shown in Fig. 10. In cells treated with DEA the area to which UL31 was recruited was much larger compared to those formed in cells treated with DMSO. To quantify these results a ratio of areas was calculated (Area^Recruit^/Area^Rad^). We define the ratio of areas as the area of UL31 preferential recruitment at its maximum (Area^Recruit^) divided by the area of the region of interest where microirradiation was directed (Area^Rad^) (Fig. 10B). In UL31 expressing cells treated with DEA this ratio averaged 3.85, meaning the average zone of UL31 recruitment had an area 3.85 times larger than the region that was microirradiated (Fig. 10C). In DMSO treated UL31 expressing cells the average ratio of areas was 2.6. The regions of UL31 recruitment in DEA treated cells were also observed to have irregular margins when compared to sites of UL31 recruitment in DMSO treated cells. A similar trend was also observed with the PAR-binding protein, H2A1.1 macro domain, with a significantly larger ratio of areas observed in DEA treated cells compared to DMSO treated cells. NBS-1 on the other hand, showed no significant increase in the ratio of areas between treated and untreated cells, indicating the increase in UL31 recruitment area caused by DEA was specific to PAR-binding proteins. These findings further support the idea that UL31 is recruited to DNA damage in a PAR-dependent manner.

**Figure 11:**
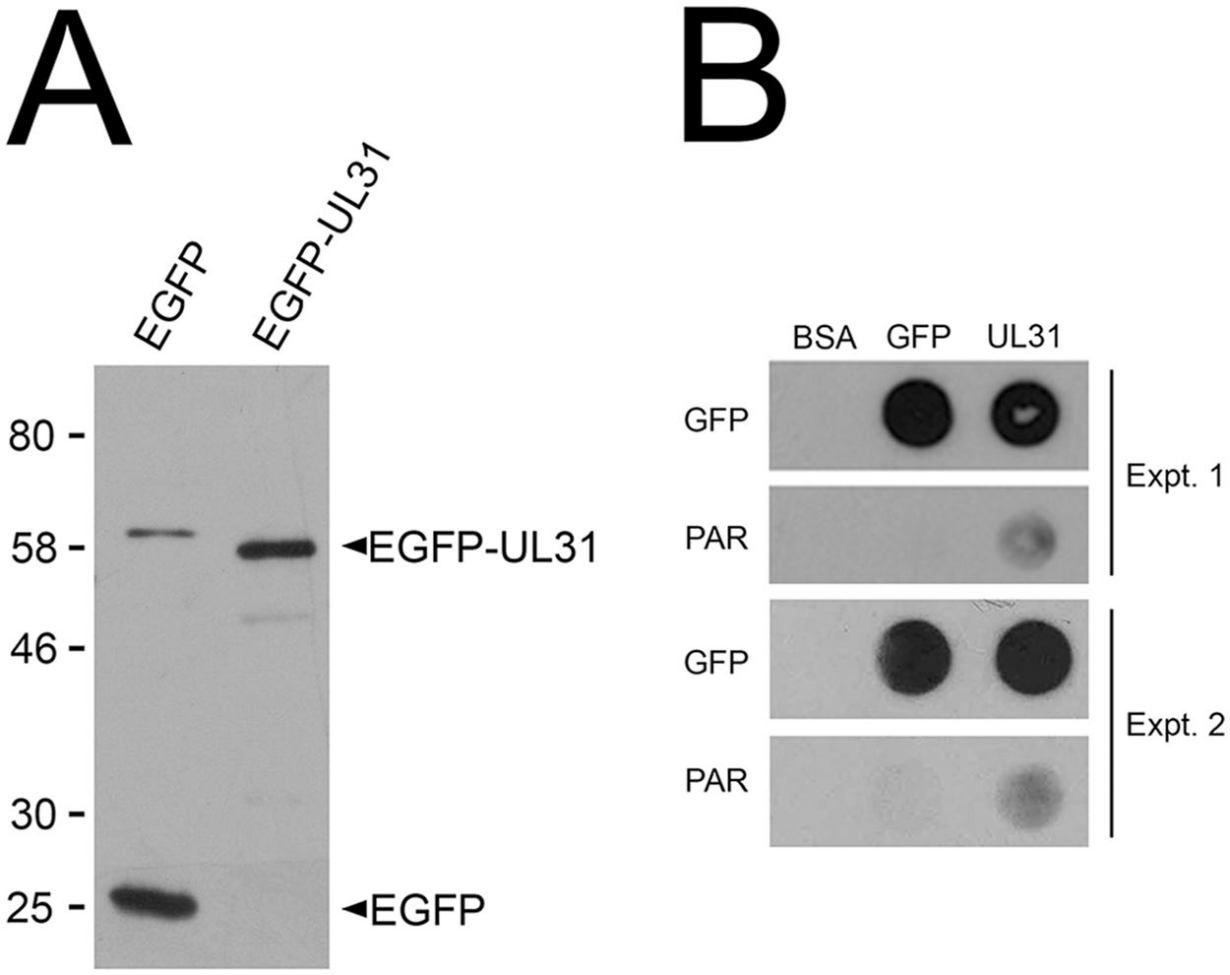
UL31 binds directly to PAR. (**A**) Western blot of GFP-TRAP purified EGFP and EGFP-UL31 probed with a GFP monoclonal antibody. Migration of molecular weight markers (in kDa) are shown on the left of the blot. The approximately 60 kDa species found in the EGFP lane likely represents an EGFP dimer that was not denatured prior to, or during, electrophoresis. The blot has been cropped above 80 kDa and below 25 kDa. The full length blot is provided as supplementary information. (**B**) Purified BSA, EGFP and EGFP-UL31 were spotted onto PVDF membranes in duplicate. One sample was probed for EGFP using a GFP monoclonal antibody and the other was first incubated in PAR polymer and subsequently probed for PAR using anti-PAR antibodies. The results from two independent experiments are shown.

To determine if UL31 was capable of binding to PAR directly, unfused EGFP and EGFP-UL31 proteins were affinity purified from cells stably expressing these proteins (Fig. 11A). Purified BSA, EGFP and UL31-EGFP were spotted in duplicate onto PVDF membranes and either probed using EGFP antiserum, or incubated with purified PAR followed by anti-PAR antiserum^38^. The results of two independent experiments are shown in Fig. 11B and indicate that EGFP-UL31 was capable of binding PAR directly whereas unfused EGFP and BSA were not.

### Recruitment of UL31 to sites of DNA damage is conserved between diverse herpesviruses

Humans are the natural host of eight distinct herpesviruses. HSV-1 and HSV-2 as well as varicella-zoster virus are members of the alphaherpesvirinae subfamily, human cytomegalovirus (HCMV) along with human herpesvirus (HHV) 6 and HHV7 belong to the betaherpesvirinae subfamily and Kaposi’s sarcoma herpesvirus (KSHV) and Epstein-Barr virus (EBV) are in the gammaherpesvirinae subfamily. To investigate whether the PAR dependent recruitment of HSV-2 UL31 to sites of laser microirradiation was conserved between Herpesviridae subfamilies, *ORF69* and *UL53*, the *UL31* orthologs from KSHV and HCMV, respectively, were fused to EGFP and tested for PARP1/2 dependent recruitment to sites of laser microirradiation (Fig. 9). Whereas there was little evidence of UL53 recruitment, ORF69 demonstrated significant recruitment to sites of laser microirradiation that was sensitive to olaparib and therefore dependent upon PARP1/2 activity.

### UL31 is recruited to sites of laser microirradiation in HSV-2 infected cells

**Figure 12:**
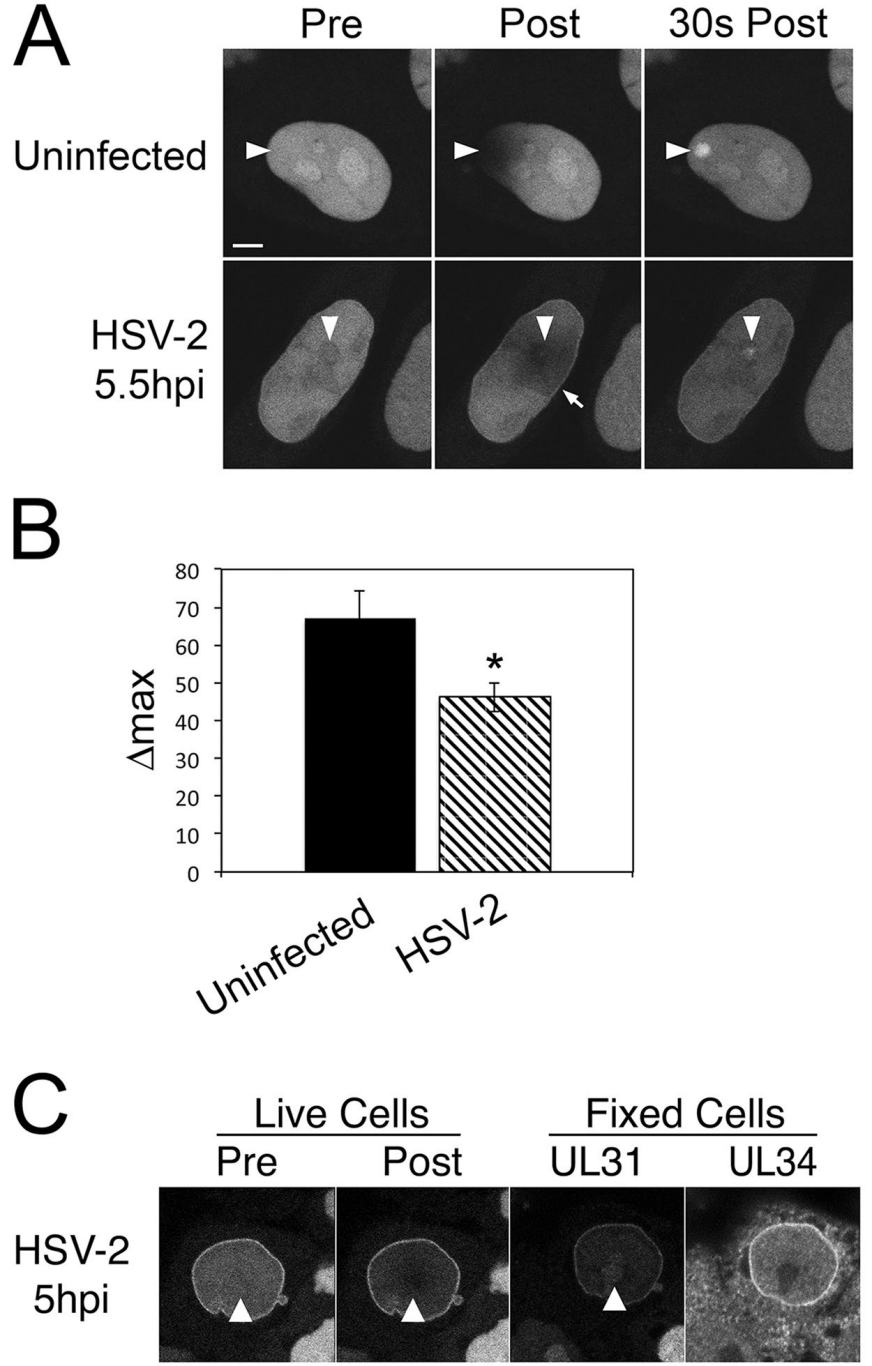
UL31 is preferentially recruited to sites of laser microirradiation in HSV-2 infected cells. (**A**) HeLa/EGFP31 cells were either mock-infected (uninfected) or infected with HSV-2 at an MOI of 3 for 5.5 h prior to microirradiation and time lapse analysis. Representative images from time lapse experiments before irradiation (Pre), immediately after irradiation (Post) and 30 s post irradiation are shown. Arrowheads indicate sites of laser microirradiation. Arrow indicates concentration of EGFP-UL31 at the nuclear rim, a surrogate indicator of UL34 expression and virus infection. (**B**) Quantitative analysis of UL31 recruitment to sites of laser microirradiation. n = 19 for infected cells in two independent experiments, n = 5 for uninfected cells. Using an unpaired *t* test the Δmax of UL31 in uninfected HeLa/EGFP31 cells was compared to the Δmax of HSV-2 infected HeLa/EGFP31 cells (*p < 0.05). (**C**) Images of a live HeLa/EGFP31 cell on a grid-bottom dish and infected with HSV-2 for 5 hours. Images taken immediately before (Pre), and immediately after (Post) the indicated area (white arrowhead) was microirradiated with a 405 nm laser using an Olympus FV1000 laser-scanning confocal microscope. Cells were immediately fixed and stained with anti-UL31 antibody followed by staining with Alexa 568 conjugated donkey anti-chicken secondary antibody. Microirradiated cells were relocated and images captured by confocal microscopy (Fixed Cells). Image is representative of n = 8 cells analyzed in two independent experiments.

Finally, we determined if UL31 had the capacity to be recruited to sites of laser microirradiation in virally infected cells. Testing this capacity in infected cells was challenging for two technical reasons. Firstly, a consequence of HSV-2 infection is the margination of cellular chromatin to the periphery of the infected cell nucleus beginning around 6 hours post infection. This complicates our ability to identify regions within the infected cell nucleus that will contain chromatin available for laser-induced damage. Secondly, prior to 6 hours post infection, little UL31 has been synthesized by the infected cell thereby obfuscating our ability to detect it. To overcome these challenges we constructed a HeLa cell line that stably expresses EGFP-UL31, HeLa/EGFP31. Uninfected HeLa/EGFP31 cells behaved similarly to HeLa cells transfected with the EGFP-UL31 plasmid after laser microirradiation (Fig. 12A,B). HeLa/EGFP31 cells infected with HSV-2 at a multiplicity of infection of 3 for 5.5 h demonstrated recruitment of EGFP-UL31 to the nuclear periphery indicative of UL34 expression (arrow in Fig. 12A) and thus virus infection. Upon laser microirradiation of infected cell nuclei, EGFP-UL31 was preferentially recruited to irradiated foci to a modestly, yet reproducibly, lower degree than that seen in uninfected cells (Fig. 12B). Considering that Us3 activity can prevent UL31 recruitment to sites of DNA damage (Fig. 6A), it may be that Us3 expression in the virally infected cells contributes to the reduction in UL31 recruitment to sites of microirradiation in infected versus uninfected cells. While a multiplicity of infection of 3 was used to ensure infection of all cells, we additionally performed correlative microscopy by fixing cells immediately after laser microirradiation, staining them for UL34 expression and relocating them for image acquisition to confirm that the irradiated cells were infected (Fig. 12C). These findings indicate that EGFP-UL31 can be recruited to sites of DNA damage in HSV-2 infected cells.

## Discussion

We have identified sequences in the N-terminus of HSV-2 UL31 that are necessary and sufficient for PARP1/2 dependent recruitment to sites of DNA damage. These observations are consistent with PAR binding activity of full length UL31 (Fig. 11B) and the presence of several putative PAR binding motifs (PBM), HKR/X_(2)_/AVIQY/KR/KR/AVIL/VILFP, within the N-terminus of the molecule^35^. We determined that residues 12–20, 21–23, 27–45 and 54–63 all contributed to UL31 recruitment to sites of microirradiation (Fig. 5D). Each of these regions contains multiple basic residues that partially resemble a canonical PBM with the closest being residues 37–44 (RASRKSLP).

The N-terminus of UL31 contains sequences that are important for UL31 function^16^. Work from the Baines laboratory indicated that serine residues in the N-terminus of HSV-1 UL31 serve as substrates for the viral Us3 kinase and that mutation of these serines to alanine resulted in reduced virus replication kinetics. However, deletion of codons 2–44 from UL31 was unable to complement a UL31 null virus suggesting the N-terminus of UL31 also contributes to UL31 function independently of its regulation by Us3. We speculate that the ability of UL31 to bind PAR through its N-terminus is a critical activity and that loss of these sequences resulted in the failure of the 2–44 deletion to complement the UL31 null strain. Interestingly, Blaho and colleagues accurately predicted that HSV-1 UL31 would be modified by nucleotidylation based on the presence of the sequence, RRASR, found in residues 37–41 of HSV-1 UL31 and in residues 36–41 of HSV-2 UL31^39^. This sequence overlaps the putative PBM identified in HSV-2 UL31. While the significance of UL31 nucleotidylation remains to be determined and the site of nucleotidylation has not been identified, these findings further highlight the importance of the N-terminal region of UL31. More recently, Funk and colleagues have reported an analysis of the N-terminal sequences of HSV-1 UL31 and concluded that this region contains a bipartite NLS that is not absolutely required for UL31 localization, is essential for HSV-1 propagation and regulates the ability of UL31 to interact with UL34^15^. Additionally, these authors suggested that the N-terminus of HSV-1 UL31 is critical for the recruitment of nucleocapsids from the nucleoplasm to sites of primary envelopment at the inner nuclear membrane.

An analysis of PRV UL31 from the Mettenleiter group identified N-terminal sequences required for its nuclear localization^40^. Substitution of four arginine residues for alanine in this region resulted in predominantly cytoplasmic localization of the protein. Additionally, this mutant failed to completely complement a UL31 null virus when provided *in trans*. By contrast, cells expressing an N-terminal truncation of 26 residues not only failed to complement the UL31 null virus, but also inhibited growth of the WT parental strain suggesting that this mutant is a dominant negative allele. The authors of this study suggested that the inhibitory effect of the truncation mutant might be due to diversion of UL34 to cytoplasmic membranes by restriction of UL31 to the cytoplasm. While this is a most reasonable hypothesis, in light of the findings presented here, we might expect that uncoupling a critical PAR binding activity from the remainder of the UL31 molecule could also result in a dominant negative phenotype. In support of this latter idea, we also found that PRV UL31 was capable of olaparib-dependent recruitment to sites of laser microirradiation (data not shown) and sequences resembling a PBM are located in the PRV UL31 N-terminus.

Consistent with the analysis of PRV UL31^40^, we determined that sequences required for HSV-2 UL31 nuclear localization are contained within its N-terminal 45 residues (Fig. 5B) and that the N-terminal 63 residues of UL31 are sufficient to restrict an EGFP fusion protein to the nucleus (Fig. 4). The conservation of a functional NLS within the N-terminus of UL31 orthologs has also been demonstrated for M53 from MCMV and UL53 from HCMV^29, 41^. A previous study concluded that residues 44–100 were critical for HSV-2 UL31 nuclear localization and that a mutant lacking residues 1–43 localized predominantly to the nucleoli of transfected cells^42^. These findings are in stark contrast to the results presented here. The reasons for these discrepancies are not clear as the overall design used for the two studies was similar and identical HSV-2 UL31 genes were used. A key difference between the two analyses was that a quantitative analysis of UL31 localization in living cells is demonstrated here, in contrast to the qualitative analyses done in fixed cells presented in the previous report. We have noted a difference in the localization pattern of EGFP-UL31 in living cells versus fixed cells (Figs 1B, 3 and 7A) insofar as the nuclei of living cells appear to contain more soluble/diffuse EGFP-UL31 that is absent (possibly extracted) from fixed cells where nucleolar localization is more prominent.

It is intriguing that UL31 has the capacity to bind to PAR because recent studies suggest that PARylation is both stimulated and stabilized in HSV-1 infected cells^43^. Metabolomics studies of HSV-1 infection demonstrated a precipitous decline in cellular NAD+ levels in HSV-1 infected cells that required viral DNA replication^43, 44^. The drop in NAD+ levels was accompanied by robust PARylation of proteins in infected cells and could be prevented by pharmacological inhibition of PARP1/2^43^. Notably, the HSV-1 ICP0 protein promotes proteasome-mediated degradation of the 111 kDa PARG isoform^43^, which would be predicted to prolong the stability of PARylated species in infected cells. While PARylation was mainly due to the activities of PARP1/2, specific inhibition of these enzymes had no measurable effect on HSV-1 replication. However, it is noteworthy that the replication of a variety of herpesviruses is influenced by PARPs. Li and colleagues demonstrated that the PARP activities of tankyrase (TNKS)1 and TNKS2 are required for efficient HSV-1 replication^45^, whereas global inhibition of PARP activity correlated with increased latent virus genome replication for the gammaherpesviruses EBV and KSHV^46, 47^.

HSV infection activates multiple cellular DNA damage responses, some of which appear to be detrimental to virus infection (e.g. DNA-activated protein kinase mediated non-homologous end joining) whereas others are beneficial for virus replication^48^. HSV modulates these cellular responses to damaged DNA by stimulating some responses and disarming others. Virus DNA replication results in complex, branched, concatemers of virus genomes containing many nicks and gaps that can be recognized by the cellular DNA damage response machinery including PARP1/2^49–51^. Interestingly, the recent work of Dembowski and DeLuca revealed that both PARP1 and UL31 are enriched on newly replicated HSV-1 DNA^19^. The HSV-1 proteins UL12 and ICP8 have been proposed to cooperate with components of the MRN complex to process these replication products, via homologous recombination, into forms competent for packaging into capsids^52, 53^. As UL31 deletion mutants demonstrate defects in genome packaging^17^, it might be that UL31 recruitment to damaged virus DNA contributes to the processing of replicated virus DNA into a form competent for packaging into capsids.

Prior to understanding that UL31 could bind PAR, we were perplexed by the discoveries that N-terminal deletions of UL31 failed to localize to nucleoli despite their significant nuclear localization (Fig. 2) and that co-expression of Us3 with UL31 also prevented UL31 nucleolar localization (Fig. 6). Moreover, fusion of the N-terminal 63 residues of UL31 was able to direct EGFP to nucleoli (Fig. 4). Thus, the ability of UL31 to localize to nucleoli correlated directly with its ability to be recruited to sites of laser microirradiation. The simplest explanation for this is that nucleoli, which are sites of active PARP1 mediated PAR synthesis^54^, recruit UL31 via its N-terminus and that Us3 can suppress this activity. How the presence of UL31 at nucleoli might influence nucleolar function or viral infection is unclear at present.

We demonstrated PARP1/2 dependent recruitment of the HSV, PRV and KSHV UL31 orthologs to sites of DNA damage but not the HCMV ortholog, suggesting that PAR binding activity is widely, but not exclusively, conserved (Fig. 9). A possible clue to why HCMV might lack this function comes from metabolomics studies on HCMV infected cells^44, 55^. Unlike the 10-fold decline in NAD+ levels observed in HSV-1 infected cells that corresponded with increased PARP1/2 dependent PARylation, the levels of NAD+ did not change substantially over the course of HCMV infection^44^. As PARP1/2 are major consumers of NAD+, these findings may suggest that these enzymes are not activated and that PAR is less abundant throughout HCMV infection. Interestingly, significant reductions in cellular NAD+ levels were observed 48 h after KSHV infection^56^.

The structures of UL31/UL34 complexes from both HSV-1 and PRV have recently been reported^57, 58^. While the solution of these structures represents an important achievement providing new understanding of the function of UL31 and UL34 in nuclear egress, the N-terminal 50 residues of HSV-1 were not present in the complex. This was unfortunate as structural information about the HSV UL31 N-terminus would have been helpful in the interpretation of the findings presented here. Several distinct activities have been ascribed to UL31 orthologs including roles in DNA replication, packaging of DNA into the capsid, nuclear egress of DNA containing capsids and activation of the transcription factor NF-kB. How UL31 binding to PAR might facilitate one or more of these activities is uncertain at present and will require the phenotypic analysis of mutant HSV-2 strains that specifically lack UL31 PAR binding function. As our data suggest that UL31 nuclear localization and PAR-binding activity are inextricably linked and Us3 phosphorylation sites on UL31 that regulate its activity and nucleolar localization are also embedded within these sequences, the isolation of such mutants is not expected to be straightforward.

## Methods

### Cells and Viruses

Vero, HaCaT, L/EGFP, HeLa and HeLa/EGFP-UL31 cells were maintained in Dulbecco’s modified Eagle’s medium (DMEM) supplemented with 10% fetal calf serum (FCS) and grown at 37 °C in a 5% CO_2_ environment. HeLa/EGFP-UL31 were constructed by transfecting HeLa cells with linearized plasmid encoding EGFP-UL31 and selecting for G418 resistant cells in medium containing 400 μg/ml G418. G418 resistant cells stably expressing EGFP-UL31 were identified and isolated with the aid of a Nikon TE200 inverted epifluorescence microscope and were maintained in medium supplemented with G418. HSV-2 strain HG52 was propagated on Vero or HaCaT cells.

### Plasmids

The various HSV-2 UL31 or UL31 ortholog expression plasmids used in this study were constructed by amplifying sequences of interest by PCR using the primers and templates described in Table 1. For construction of EGFP-UL31, EGFP-ORF69, EGFP-UL53 and truncated EGFP-UL31 versions ∆11, ∆20, ∆23, ∆27, ∆45 and N63, *Eco*RI and *Sa*lI cleavages sites were engineered into the forward and reverse primers, respectively. Amplified products were digested with *Eco*RI and *Sal*I and ligated into similarly digested pEGFP-C1 (Clontech Laboratories, Mountain View, CA). For construction of truncated EGFP-UL31 versions 12–63, 21–63, 12–54, 21–54, a forward primer complementary to the multiple cloning site of pEGFP-C1 was paired with a reverse primer containing either a *Sal*I (12–63 and 21–63) or a *Bam*HI (12–54 and 21–54) cleavage site. Amplified products were digested with *Eco*RI and *Sal*I or *Bam*HI and ligated into similarly digested pEGFP-C1. To delete conserved region 1 (∆CR1) from EGFP-UL31, splice overlap extension PCR^59^ was used to generate a product lacking residues 59 to 126, which was then digested with *Eco*RI and *Sal*I and ligated into similarly digested pEGFP-C1. To construct FLAG-UL34, PCR utilizing a forward primer 5′-AGTTCGAATTCTATGGCGGGGATGGGGAAGCCCTACG-3′ and reverse primer 5′-GATCGTCGACTCATATAGGCGCGCGCCAACCGCC-3′ were used to amplify the UL34 gene using purified HSV-2 DNA as template. The product was digested with *Eco*RI and *Sal*I and ligated into similarly digested pFLAG-CMV-2 (Sigma Aldrich, St. Louis, MO). All plasmids constructed utilizing PCR were sequenced to ensure that no spurious mutations were introduced. Craig McCormick and Eric Pringle, Dalhousie University, provided KSHV strain rKSHV.219 DNA and Karen Mossman, McMaster University, provided HCMV strain AD169 DNA that served as templates for the construction of EGFP-ORF69 and EGFP-UL53 expression constructs, respectively. The pNbs1-2GFP expression plasmid was provided by Jiri Lukas, University of Copenhagen^33^ and plasmid pDEST47 containing the gene for the macro domain H2A1.1 fused to EGFP was provided by Andrew Jefferson and Ivan Ahel, University of Oxford^60^.

**Table 1:** Primers used to construct UL31 expression plasmids.

Plasmid	Forward Primer (5′-3′)	Reverse Primer (5′-3′)	Template
EGFP-UL31	AGTTCGAATTCTATGTATGACATCGCCCCCCGTCGC	GATCGTCGACCTACGGCGGAGGAAACTCGTCG	HSV-2 HG52 vDNA
Δ11	GATCGAATTCTCGGCCCGGGCCCGGCCGCGACAAG	GATCGTCGACCTACGGCGGAGGAAACTCGTCG	EGFP-UL31
Δ20	GATCGAATTCTCGGCGGCG GTCGCGCTTTTCC	GATCGTCGACCTACGGCGGAGGAAACTCGTCG	EGFP-UL31
Δ23	GATCGAATTCTTCGCGCTTT TCCGCCGCCGGGAACC	GATCGTCGACCTACGGCGGAGGAAACTCGTCG	EGFP-UL31
Δ27	GATCGAATTCTGCCGCCGG GAACCCCGGCGTG	GATCGTCGACCTACGGCGGAGGAAACTCGTCG	EGFP-UL31
Δ45	GACTGAATTCTCACGCGCGCAGGCTGGAGCTCTGCCTGCACG	GATCGTCGACCTACGGCGGAGGAAACTCGTCG	EGFP-UL31
N63	AGTTCGAATTCTATGTATGACATCGCCCCCCGTCGC	GATCGTCGACCTAGAAGCCGCGGTAGCGACG	EGFP-UL31
12–63	GCAGAGCTGGTTTAGTGAACC	GATCGTCGACCTAGAAGCCGCGGTAGCGACG	Δ11
21–63	GATCGAATTCTCGGCGGCGGTCGCGCTTTTCC	GATCGTCGACCTAGAAGCCGCGGTAGCGACG	Δ20
12–54	GCAGAGCTGGTTTAGTGAACC	GATCGGTACCTTACAGGCAGAGCTCCAGCCTGC	Δ11
21–54	GATCGAATTCTCGGCGGCGGTCGCGCTTTTCC	GATCGGTACCTTACAGGCAGAGCTCCAGCCTGC	Δ20
ΔCR1 5′	AGTTCGAATTCTATGTATGACATCGCCCCCCGTCGC	GGCGCGGTAGCGACGGCG CTCGTGCAG	EGFP-UL31
ΔCR1 3′	CCTGCACGAGCGCCGTCGCTACCGCGGCAGCGCGGGCGACGGGCGGCTGGCCACC	GATCGTCGACCTACGGC GGAGGAAACTCGTCG	EGFP-UL31
HCMV UL53	GATCGAATTCTATGTCTAGCGTGAGCGGCGTGC	GATCGGTACCTCAGGGCGCACGAATGCTCTTG	HCMV vDNA
KSHV ORF69	AGTTCGAATTCTATGCC GAAATCAGTGTCCAGCC	GATCGTCGACTTATAGGGCGTTGACAAGTGC	KSHV iSLK.219 infected cells

### Inhibitors

To inhibit ataxia telangiectasia mutated (ATM) kinase, HeLa cells were treated with 10 µM KU-55933 (EMD Millipore, Billerica, MA) 2 hours prior to imaging experiments. To inhibit PARP1/2, HeLa cells were treated with 10 nM olaparib (LC Laboratories, Woburn, MA) one hour prior to imaging experiments. PARG was inhibited using 6,9-diamino-2-ethoxyacridine lactate monohydrate (DEA) (Sigma Aldrich, St. Louis, MO) at a concentration of 1 µM one hour prior to imaging experiments. Control samples in these experiments received an equivalent amount of DMSO (0.1% final).

### Immunological reagents

For indirect immunofluorescence analysis, mouse anti-phospho-Histone H2A.X monoclonal antibody (EMD Millipore, Billerica, MA) was used at a dilution of 1:500; rabbit anti-ATM (phospho S1981) monoclonal antibody (Abcam, Cambridge, MA) was used at a dilution of 1:100; mouse anti-PAR polymer Clone 10H monoclonal antibody (Tulip Biolabs, Lansdale, PA) was used at a dilution of 1:100; mouse anti-FLAG epitope monoclonal antibody (Sigma, Oakville, ON) was used at a dilution of 1:80; Alexa Fluor 568 conjugated donkey anti-mouse, Alexa Fluor 568 conjugated donkey anti-goat, and Alexa Fluor 647 conjugated donkey anti-mouse (Molecular Probes, Eugene, OR) were all used at a dilution of 1:500. For Western blot and dot blot analyses mouse monoclonal EGFP antibody (Clontech Laboratories, Mountain View, CA) was used at a dilution of 1:1,000; mouse anti-PAR polymer Clone 10H monoclonal antibody were used at dilutions of 1:100; horseradish peroxidase (HRP)-conjugated goat anti-mouse (Sigma, Oakville, ON) were used at a dilution of 1:10,000. Chicken antisera was raised by Cedarlane Laboratories (Burlington, ON) against a GST-HSV-2 UL34 fusion protein expressed in, and purified from *E*. *coli*.

### Indirect immunofluorescence microscopy

Cells grown on glass bottom dishes were fixed in 4% para-formaldehyde/PBS at room temperature for 15 minutes and processed for indirect immunofluorescence microscopy as described previously^61^. Images of stained cells were acquired using an Olympus FV1000 confocal microscope using a 60X (1.42 NA) objective and Fluoview software version 1.7.3.0. Composites of representative images were prepared using Adobe Photoshop software.

### Laser microirradiation

HeLa cells growing in glass bottom dishes (MatTek, Ashland, MA) were transfected with a total of one µg of plasmid DNA using X-tremeGENE HP DNA transfection reagent according to the manufacturer’s instructions (Roche, Laval, QC). Sixteen hours after transfection, the medium was replaced with warm DMEM (lacking phenol red)/10% FCS and the cells were mounted onto an Olympus FV1000 confocal microscope and maintained at 37 °C in a humidified 5% CO_2_ environment. Transfected cells were identified using a 60X (1.42 NA) oil immersion objective and a 488 nm laser line set at 5% power. Images (512 by 512 pixels with a digital zoom factor of 6) were collected at a rate of 0.33 frames per second, except for the data shown in Fig. 8 where images were collected at a rate of 0.2 frames per second. Five frames were collected before a defined region of the imaged cells was microirradiated by repeated scanning with a 405 nm laser set at 100% power for a total of 3000 ms. Before, during, and after microirradiation, fluorescence intensities in irradiated and non-irradiated control regions were measured using Olympus Fluoview software version 1.7.3.0. Data were exported into Microsoft Excel for graphical presentation and composites of representative images were prepared using Adobe Photoshop. Similar procedures were used for the analysis of HSV-2 infected HeLa/EGFP-UL31 cells.

### Correlative microscopy

Cells growing on glass bottom dishes etched with an alphanumeric grid (MatTek, Ashland, MA) were transfected as described above. Laser microirradiation assays were then performed on 4–6 cells within a specific quadrant of the grid. These experiments used the same parameters as in the laser microirradiation assays described above with the exception that three frames were collected before microirradiation, at a rate of one frame per second, with a total of 5 frames being captured. Immediately after the last cell was microirradiated, cells were washed twice with PBS then fixed in 4% para-formaldehyde/PBS in preparation for indirect immunofluorescence microscopy.

### Determination of nuclear:cytoplasmic ratio and Δmax

Using an Olympus FV1000 confocal microscope and Fluoview software version 1.7.3.0, the fluorescence intensity of defined regions within the nuclei and cytoplasm of transfected cells were measured and used to calculate nucleus to cytoplasm ratios (N/C). To gauge the ability of recombinant EGFP proteins to be recruited to sites of DNA damage during microirradiation experiments, Δmax values were calculated. Δmax is the maximum positive difference between the fluorescence intensity of the microirradiated region and a control region within a cell nucleus. Comparisons of N/C and Δmax values were made utilizing unpaired *t* tests with unequal variance.

### Purification and analysis of EGFP fusion proteins

A GFP-Trap kit (Chromotek, Hauppauge, NY) was used to purify EGFP and EGFP-UL31 from L/EGFP cells or HeLa/EGFP-UL31, respectively. Cells growing on 150 mm dishes were washed once with 20 ml cold PBS then scraped into 3 ml of cold PBS. Cellular lysates were prepared and proteins were purified according to the manufacturer’s instructions. Proteins in samples were separated by SDS-PAGE and then transferred to polyvinylidene fluoride (PVDF) membranes (Millipore, Billerica, MA) that were subsequently blocked with Tris-buffered saline (TBS) containing 0.05% Tween 20 (TBST) and 3% bovine serum albumin (BSA). After blocking, the membranes were probed with appropriate dilutions of primary antibody followed by appropriate dilutions of horseradish peroxidase-conjugated secondary antibody, and proteins were detected using Pierce ECL Western blotting substrate (Thermo Scientific, Rockford, IL) and exposure to film. For dot blot analyses^38^, purified proteins were diluted into a volume of 170 µl of TBST and transferred onto a PVDF membrane in duplicate using a vacuum manifold. The samples were air-dried, washed three times in TBST followed by a one-hour incubation in TBST. One set of samples was incubated with 250 nM PAR polymer (Amsbio, Cambridge, MA) for one hour at room temperature, washed 3 times in TBST then both sets of samples were blocked in TBST/3% BSA for 1 hour at room temperature. Blocked membranes were incubated with primary antibody (EGFP or PAR monoclonal antibodies) and horseradish peroxidase-conjugated secondary antibody diluted appropriately in TBST/1% BSA and reactive antibodies visualized using Pierce ECL Western blotting substrate and exposure to film.

## Electronic supplementary material

Supplementary Information
