# Supplementary material for: The Herpesvirus Nuclear Egress Complex Component, UL31, Can Be Recruited to Sites of DNA Damage Through Poly-ADP Ribose Binding
Source: Sci Rep. 2017 May 15;7:1882. doi: 10.1038/s41598-017-02109-0 (PMC5432524; doi:10.1038/s41598-017-02109-0)

Supplementary Information

Title: The Herpesvirus Nuclear Egress Complex Component, UL31, Can Be Recruited to Sites of DNA Damage Through Poly-ADP Ribose Binding

Authors: Maxwell R. Sherry, Thomas J. M. Hay, Michael A. Gulak, Arash Nassiri, Renée L. Finnen and Bruce W. Banfield

Figure 11A of the manuscript shows a western blot probed for EGFP that was cropped above the 80kDa marker and below the 25kDa marker. The image has been flipped vertically from the original scan and cropped to remove the lane containing the degraded N63 sample that was not used in further analyses. The full-length blot is shown below.

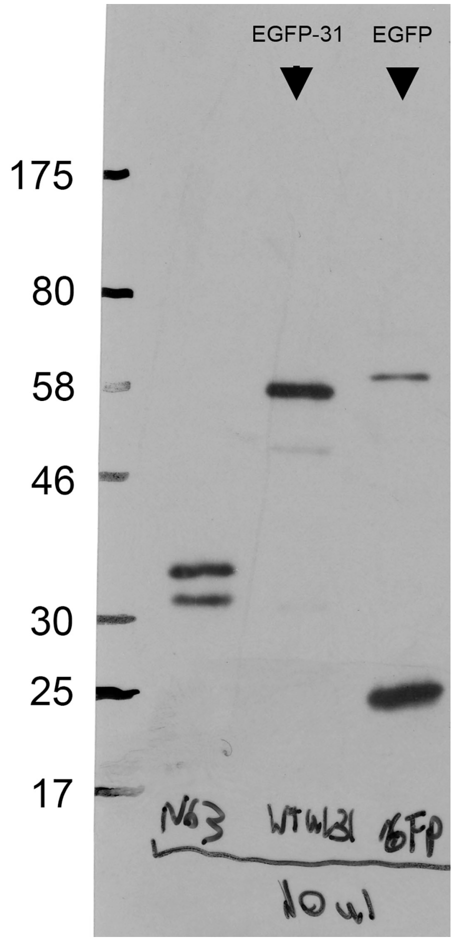

Supplement: Supplementary file 1 — Supplementary Information [file 41598_2017_2109_MOESM1_ESM.pdf]
